# Anti-Inflammatory Effects of Rosmarinic Acid-Loaded Nanovesicles in Acute Colitis through Modulation of NLRP3 Inflammasome

**DOI:** 10.3390/biom11020162

**Published:** 2021-01-26

**Authors:** Sonia Marinho, Matilde Illanes, Javier Ávila-Román, Virginia Motilva, Elena Talero

**Affiliations:** 1 Health Sciences Center, Federal University of Recôncavo da Bahia, Santo Antônio de Jesus 44430-400, Brazil; soniamarinho@ufrb.edu.br; 2 Department of Normal and Pathological Cytology and Histology, Universidad de Sevilla, 41009 Seville, Spain; alamo110@us.es; 3 Department of Biochemistry and Biotechnology, Faculty of Chemistry, Universitat Rovira i Virgili, 43007 Tarragona, Spain; franciscojavier.avila@urv.cat; 4 Department of Pharmacology, Faculty of Pharmacy, Universidad de Sevilla, 41012 Seville, Spain; motilva@us.es

**Keywords:** ulcerative colitis, rosmarinic acid, nanovesicles, inflammasome, Nrf2

## Abstract

Ulcerative colitis (UC), one of the two main types of inflammatory bowel disease, has no effective treatment. Rosmarinic acid (RA) is a polyphenol that, when administered orally, is metabolised in the small intestine, compromising its beneficial effects. We used chitosan/nutriose-coated niosomes loaded with RA to protect RA from gastric degradation and target the colon and evaluated their effect on acute colitis induced by 4% dextran sodium sulphate (DSS) for seven days in mice. RA-loaded nanovesicles (5, 10 and 20 mg/kg) or free RA (20 mg/kg) were orally administered from three days prior to colitis induction and during days 1, 3, 5 and 7 of DSS administration. RA-loaded nanovesicles improved body weight loss and disease activity index as well as increased mucus production and decreased myeloperoxidase activity and TNF-α production. Moreover, RA-loaded nanovesicles downregulated protein expression of inflammasome components such as NLR family pyrin domain-containing 3 (NLRP3), adaptor protein (ASC) and caspase-1, and the consequent reduction of IL-1β levels. Furthermore, nuclear factor erythroid 2-related factor 2 (Nrf2) and heme oxygenase-1 (HO-1) protein expression increased after the RA-loaded nanovesicles treatment However, these mechanistic changes were not detected with the RA-free treatment. Our findings suggest that the use of chitosan/nutriose-coated niosomes to increase RA local bioavailability could be a promising nutraceutical strategy for oral colon-targeted UC therapy.

## 1. Introduction

Inflammatory bowel disease (IBD), comprising UC and Crohn’s disease (CD), is characterised by chronic and relapsing intestinal inflammation. Accumulating evidence indicates that these inflammatory disorders are multifactorial, triggered by interactions between genetic, environmental and immune factors [1]. Although the etiology of IBD is still unclear, an aberrant innate immune response against internal and external threatening factors has been suggested to have a crucial role in IBD pathogenesis [1]. Inflammasome is a cytosolic multiprotein complex involved in the innate immune response, which is stimulated by many types of tissue damage. NLRP3 is one of the best representative inflammasomes, which has been associated with UC pathogenesis [2,3]. NLRP3 stimulation leads to proteolytic activation of caspase-1, which is crucial for the cleavage of pro-IL-1β into its active form. Mature IL-1β is a key inflammatory mediator that participates in the inflammation occurring in IBD patients, and its overproduction is related to augmented disease severity [4].

In the inflamed colon, activated macrophages and neutrophils lead to overproduction of reactive oxygen species (ROS). Nrf2 is a key factor in the protection against oxidative stress and inflammation. Upon oxidative stress, Nrf2 induces nuclear transcription of endogenous antioxidant enzymes, such as HO-1 [5]. Activation of the Nrf2/HO-1 pathway has been reported to attenuate the inflammatory response in experimental colitis [6], while suppressing Nrf2 increased inflammation and oxidative stress [7]. Thus, pharmacological induction of this pathway may be a useful strategy for IBD treatment.

It has been reported that dextran sodium sulfate (DSS) administration to mice in drinking water induces a very reproducible acute inflammation limited to the colon. This model is widely used in mice for inducing UC since it morphologically and symptomatically resembles epithelial damage found in human patients suffering from UC [8]. In this line, DSS is a sulphated polysaccharide that acts as a direct toxin leading to the disruption of the colonic epithelium. This results in increased permeability and the entry of luminal pathogens and associated antigens into the mucosa, leading to immune cell activation with the consequent production of proinflammatory cytokines, which, in turn, aggravates epithelial barrier dysfunction [9].

Current IBD treatments include amino-salicylates, corticosteroids, immunosuppressants and, recently, biological agents, which have been effective in minimizing inflammation and inducing prolonged remission. However, these therapies fail to effectively control symptoms and have severe adverse effects [10]. For this reason, there is an urgent need to seek new therapeutic strategies for clinical prevention and treatment of IBD. In this line, dietary supplements such as omega 3 fatty acids, vitamin D and polyphenols, including curcumin and resveratrol, have been shown to have a therapeutic effect on IBD [11]. Rosmarinic acid (RA) is a natural polyphenol found in the *Labiatae* family of herbs, such as *Rosmarinus officinali*, *Salvia miltiorrhiza*, and *Prunella vulgaris*, as well as in *Zostera marina* seagrass beds [12]. RA has been shown to have many biological properties, including antioxidant, anti-inflammatory, anticancer, anti-infectious, antinociceptive and neuroprotective activities [13,14,15]. Nevertheless, when administered orally, its poor water solubility and high gastric degradation may compromise its beneficial effects [16]. In addition, previous papers have reported a high metabolization of RA in the upper gastrointestinal tract. In this regard, in vitro digestion studies have described that RA is hydrolysed by different esterases and metabolised by the gut microbiota before its absorption, which could affect the functionality of this polyphenol as reviewed in [17]. The oral administration of appropriately formulated RA would improve its bioavailability and allow an adequate RA concentration to reach the inflamed colon.

The use of nanotechnology in medicine has gained extensive attention in the delivery of drugs in inflamed intestinal mucosa. In this regard, niosomes are vesicles mostly formed by cholesterol and nonionic surfactants. Their structure is similar to liposomes and, like them, can contain both hydrophilic and lipophilic molecules. However, niosomal carriers could be used as an interesting alternative to liposomes due to their lower production cost and higher stability [18]. Colon-specific delivery strategies based on nanosystems are being used to increase drug local bioavailability and reduce dosing frequency, minimizing adverse effects. Chitosan is a natural polymer extensively used for coating nanovesicles with interesting properties including biocompatibility, biodegradability and low toxicity. Moreover, chitosan is highly mucoadhesive, which consequently increases drug absorption and promotes persistent drug release [19]. This polysaccharide administered orally can resist gastrointestinal degradation and passes unaltered to the colon where it can be partially degraded by microbiota. A previous paper evaluating the effects of quercetin in 2,4,6-trinitrobenzene sulfonic acid (TNBS)-induced colitis reported that phospholipid vesicles coated with a combination of chitosan and nutriose, a water-soluble dextrin, protected quercetin from degradation in the upper gastrointestinal tract, thus allowing its colonic release [20].

Although RA has been previously encapsulated in a niosomal gel [21], ethosomes and liposomes [22], nanoemulsion-based hydrogels, and polyethylene glycol (PEG)ylated RA-derived nanoparticles [23], there are no reports on RA-loaded niosomes and their use in IBD. Taking into account these findings, in the present study a DSS‑induced acute colitis model in mice was established to evaluate the effects of chitosan and nutriose-coated niosomes loaded with RA. Since the potential mechanisms of RA in treatment of UC are not fully understood, we also investigated the effects of this polyphenol on modulation of inflammasome and the Nrf2/HO-1 signaling pathway.

## 2. Materials and Methods

### 2.1. Preparation of Chitosan and Nutriose-Coated Niosomes Loaded with Rosmarinic Acid

Niosomes were purchased from Nanovex Biotechnologies SL (Asturias, Spain) and all reagents, including RA, were purchased from Sigma-Aldrich (St. Louis, MO, USA). A thin film hydration method was carried out for preparing niosomes either loaded or not with RA, and chitosan and nutriose-coated or uncoated, as previously described [20]. Briefly, Pronanosome Nio-N (50 mg/mL), rosmarinic acid (5 mg/mL) and vitamin E (2 mM) were dissolved in a methanol:chloroform mixture. The organic solvents were rotary-evaporated to form a dry film, which was hydrated with phosphate buffered saline (PBS) (10 mM) and vitamin C (2 mM) at 60 °C for 20 min and vortexed for 4 min. Subsequently, the obtained nanocarriers were sonicated for 30 min at 60 °C to create small unilamellar nanovesicles. To prepare chitosan-coated niosomes loaded with RA, chitosan (30 mg/mL) was dissolved in 0.1% acetic acid solution (pH 3), then this solution was added dropwise to RA-loaded niosomes under stirring at 25 °C. Later, the subsequent dispersion was added to a 5% (*w*/*v*) nutriose solution. Empty chitosan and nutriose-coated nanovesicles were prepared following the same procedure but with no RA.

### 2.2. Vesicle Characterisation

Particle size and polydispersity index (PDI) were evaluated by a dynamic light scattering method using a Zetasizer Nano ZS90 (Malvern Instruments, Worcestershire, UK). Zeta potential (ZP) was determined with the Zetasizer Nano ZS90 by means of the M3-PALS (Mixed Mode Measurement-Phase Analysis Light Scattering), based on electrophoretic mobility. For encapsulation efficiency (EE) determination, chitosan-coated or uncoated niosomes loaded with RA were dialyzed using dialysis bag with a cutoff of 10 K (MWCO, Thermo Fisher Scientific, Waltham, MA, USA) for 24 h against PBS (pH 7.4) to completely removing unloaded RA. RA content before and after separation of free drug from the encapsulated drug was quantified with reverse-phase high-performance liquid chromatography (RP-HPLC) after disruption of niosomes with methanol. EE percentage was calculated as follows: % EE = (amount of drug retained into the vesicles/total amount of drug in the sample) × 100.

### 2.3. In Vitro Release Studies

The in vitro release profile of RA from vesicles was measured in a buffered solution at pH 7.0 to simulate large intestine conditions, using the dialysis bag method [19]. In brief, 100 µL of chitosan-coated or uncoated niosomes loaded with RA or control solution (nonencapsulated RA) were placed in a dialysis bag with a cutoff of 10 K (Slide-A-Lizer MINI Dialysis units, Thermo Fisher Scientific, Waltham, MA, USA) and immersed in the dissolution medium containing 15 mL PBS (10 mM) with 300 mM NaCl (pH 7.0) at 37 °C under magnetic stirring at 100 rpm. At scheduled time intervals (1, 2, 3, 4, 5, 6, 7 and 8 h), an aliquot of the release medium was collected and equally replenished with fresh medium. RA content in the samples was analyzed by RP-HPLC. All experiments were performed in triplicate.

### 2.4. Experimental Animals

A total of 70 seven-week-old male C57BL/6 mice weighing 18–20 g were supplied by Janvier Labs (Le Genest St Isle, France) and acclimated for seven days in Animal Laboratory Centre (Animal Service, School of Pharmacy, University of Seville). They were allowed free access to a laboratory diet (Panlab, Barcelona, Spain) and water ad libitum. All experiments were carried out following the guidelines of the European Union in relation to animal experimentation (Directive of the European Council 2010/63/EU). The experimental protocols were approved by the Animal Ethics Committee of the University of Seville (Protocol 06/04/2018/042).

### 2.5. Induction of Acute Colitis and Treatments

Experimental acute colitis was induced by giving mice drinking water ad libitum containing 4% (*w*/*v*) DSS (TdB Consultancy AB, Uppsala, Sweden), for seven days [24]. After an acclimation period, mice were randomly divided into seven experimental groups (n = 10/group). Except the sham group (that consumed water), the remaining experimental groups received 4% DSS solution ad libitum. According to the experimental protocol, the following solutions were administered by oral gavage: (i) Sham and (ii) DSS groups, vehicle solution (PBS 10 mM) with empty nanovesicles; (iii) 5-aminosalicylic acid group (5-ASA), 5-ASA at 75 mg/kg/day, used as positive reference compound; (iv) RA group, free RA at 20 mg/kg/day; (v) RA-N5 group, RA-loaded nanovesicles at 5 mg/kg/day; (vi) RA-N10 group, RA-loaded nanovesicles at 10 mg/kg/day; and (vii) RA-N20 group, RA-loaded nanovesicles at 20 mg/kg/day [25]. Mice received oral pretreatment with all solutions from three days prior to colitis induction and during days 1, 3, 5 and 7 of DSS administration.

Animals were carefully monitored to verify that the consumption of water containing DSS was approximately the same in all groups. Animal body weights were measured daily throughout all the experiments. Mice were sacrificed on 8th day and the entire colon was removed, cleaned with physiological saline, weighed and measured. Subsequently, small sections from the middle to distal colon were cut and stored at −80 °C for measurement of all biochemical parameters.

### 2.6. Evaluation of Severity of Colitis

To determine the disease activity index (DAI), the clinical signs of colitis was evaluated during experimentation as previously reported [26]. The presence of diarrhea, rectal bleeding, and weight loss were independently scored by a blinded-researcher on a 0 to 3 scale, and the average of the three determinations constituted the DAI.

### 2.7. Histopathological Evaluation

For histological examination, sections of approximately 1 cm from the middle to distal colon were fixed in 4% paraformaldehyde in PBS (pH 7.4), dehydrated and embedded in paraffin. Next, samples were sectioned at 5 µm by using a rotary microtome (Leica Microsystems, Wetzlar, Germany) and mounted onto glass slides. Colon sections were dewaxed, hydrated, and stained with Haematoxylin and Eosin or Alcian blue for colonic injury examination or mucus content, respectively [27]. All samples were evaluated in an Olympus BH-2 microscope (GMI, Ramsey, MN, USA). The tissues were analysed by a blinded observer to establish a composite histological score as previously described [28]. Criteria include loss of mucosal architecture (0, absent; 1, mild; 2, moderate; 3, severe), cellular infiltration (0, none; 1, infiltrate around the crypt basis; 2, infiltrate reaching the muscularis mucosae; 3, infiltrate reaching the submucosa) and goblet cell depletion (0, absent; 1, present). The semiquantitative histopathological score of each variable was added to give a total microscopic damage score.

### 2.8. Myeloperoxidase Activity Assay

For myeloperoxidase (MPO) activity determination, colon samples were homogenized in 10 volumes of 50 mM PBS at pH 7.4, following the method of Grisham et al. (1990) [29] and then were centrifuged at 20,000 g for 20 min at 4 °C. Next, the pellet was homogenized in 10 volumes of 50 mM PBS at pH 6.0 containing 0.5% hexadecyl trimethylammonium bromide (HETAB) and 10 mM EDTA. Subsequently, samples were exposed to three cycles of freezing/thawing and later sonication. For colorimetric assay, 50 µL of homogenate were incubated at 37 °C for three min with a mixture containing 0.067% O-dianisidine dihydrochloride, 0.5% HETAB, and 0.3 mM hydrogen peroxide in a 96-well microplate. The absorbance at 655 nm was evaluated with a microplate reader (Labsystem Multiskan EX, Helsinki, Finland). One unit of MPO activity was expressed as the amount of enzyme generating a change in absorbance of 1.0 U/min at 37 °C in the final reaction volume. Data were expressed as U/mg tissue.

### 2.9. Determination of Cytokine Levels

Colon tissue samples for cytokine determination (TNF-α and IL-1β) were homogenized in ice-cold lysis buffer (1:5 *w*/*v*) containing PBS (pH 7.2), 1% bovine serum albumin (BSA) and protease inhibitors. Next, samples were centrifugated at 12,000 g for 10 min at 4 °C to obtain the supernatants, which were stored at −80 °C until determination. Cytokine concentrations were quantified using specific ELISA kits (Peprotech, London, UK), following the manufacturer’s protocol. Cytokine levels were expressed as picograms per milligram of tissue.

### 2.10. Extraction of Cytoplasmic Proteins and Western Blot Analysis

Colon samples were homogenized in lysis buffer as previously described [30] then were centrifuged (12,000 g for 15 min at 4 °C) and the supernatants stored at −80 °C. Protein concentration was quantified by Bradford´s method (Bradford, 1976) [31]. Next, equal amounts of protein (50 µg) were separated by sodium dodecyl sulphate-polyacrylamide gel electrophoresis and, subsequently, were transferred onto a nitrocellulose membrane at 120 mA for 90 min. Later, the membranes were blocked with 5% *w*/*v* BSA in PBS-Tween 20 for 1 h. After blocking, the membranes were incubated with the following primary antibodies at 4 °C overnight: rabbit anti-ASC (1:1000), rabbit anti-NLRP3 (1:1000) (Cell Signaling, Danvers, MA, USA), rabbit anti-Nrf-2 (1:500; Santa Cruz Biotechnology, Dallas, TX, USA), rabbit anti-HO-1 (1:500; Enzo Life Sciences, New York, NY, USA), and rabbit anti-Caspase-1, (1:1000 Abcam, Cambridge, UK). All the membranes were also incubated with an anti-β-actin antibody (Sigma-Aldrich, St. Louis, MO, USA) to verify equal loading. Then, the blots were washed three times for 15 min and incubated with the secondary antibody, horseradish peroxidase-linked anti-rabbit (Pierce Chemical, Rockford, IL, USA) for 60 min at room temperature. After the membranes were washed again three times, the bands were visualized using an enhanced chemiluminescence light-detecting kit (Super-Signal West Pico Chemiluminescent Substrate, Pierce, IL, USA). Densitometric analysis was performed after normalisation with the control (house-keeping gene). The signals were quantified with Scientific Imaging Systems (Biophotonics Image J Analysis Software, Bethesda, MD, USA) and plotted as a percentage in relation to DSS group.

### 2.11. Statistical Analysis

All data in the figures and text are exhibited as arithmetic means with their standard errors. Statistical analysis was carried out using SPSS statistical software (IBM SPSS Statistics version 26.0 SPSS Inc., Chicago, IL, USA). The Shapiro–Wilk test was used to verify the normality of the data. Student’s t-test was used to compare between the two control groups (sham vs. DSS). The Mann–Whitney U test was chosen for nonparametric values. Statistical differences between multiple groups were compared by one-way ANOVA followed by Bonferroni’s post hoc test for parametric data. Nonparametric data were analysed by the Kruskal–Wallis test for multiple comparisons. A *p*-value less than 0.05 was considered as statistically significant. The statistical test used for individual analyses is provided in the figure legends. For the histological study, the results presented are representative of at least five independent experiments performed on different days.

## 3. Results

### 3.1. Vesicle Characterisation

RA-loaded niosomes were prepared by the thin film hydration technique, as described in Material and Methods. Next, vesicles were coated with chitosan and nutriose for the delivery of RA to the colon. The particle size (nm), ZP (mv) and encapsulation efficiency (%) of formulations are presented in Table 1. The average diameters of niosomes were 260.7, 429.7 and 480.5 nm for uncoated niosomes loaded with RA, empty chitosan and nutriose-coated niosomes and chitosan and nutriose-coated niosomes loaded with RA, respectively. Surface charge on both uncoated and chitosan-coated niosomes was evaluated by measuring their ZP. Uncoated niosomes exhibited negative ZP values, which were inverted to positive values after coating with chitosan and nutriose, showing in an indirect manner the presence of this polymeric complex on the noisome surface.

The encapsulation efficiency of RA was high (>70%) for both uncoated and coated niosomes.

### 3.2. In Vitro Release Studies

**Figure 1:**
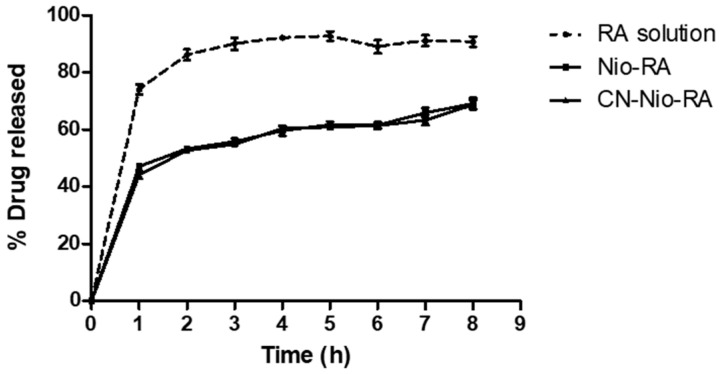
Amount of rosmarinic acid (RA) (%) released from free drug solution (RA solution), uncoated niosomes loaded with rosmarinic acid (Nio-RA), and chitosan and nutriose-coated niosomes loaded with RA (CN-Nio-RA). The study was carried out using dialysis bag method at 37 °C, and dissolution medium containing PBS (10 mM) with 300 mM NaCl (pH 7.0). All data represent mean ± SD (n = 3).

The in vitro release profiles were analysed at pH 7.0, i.e., the large intestine pH, comparing RA profile from a standard drug solution and chitosan-coated or uncoated niosomes loaded with RA (Figure 1). As regards the control, a relatively rapid release of RA was found, with 74% released within the first hour and a nearly complete release (approximately 90%) within 2 h. The amount of RA released from uncoated niosomes was similar to that obtained from chitosan-coated vesicles at each time point, exhibiting around 70% drug released after 8 h.

### 3.3. Rosmarinic Acid-Loaded Nanovesicles Protected against DSS-Induced Acute Colitis in Mice

**Figure 2:**
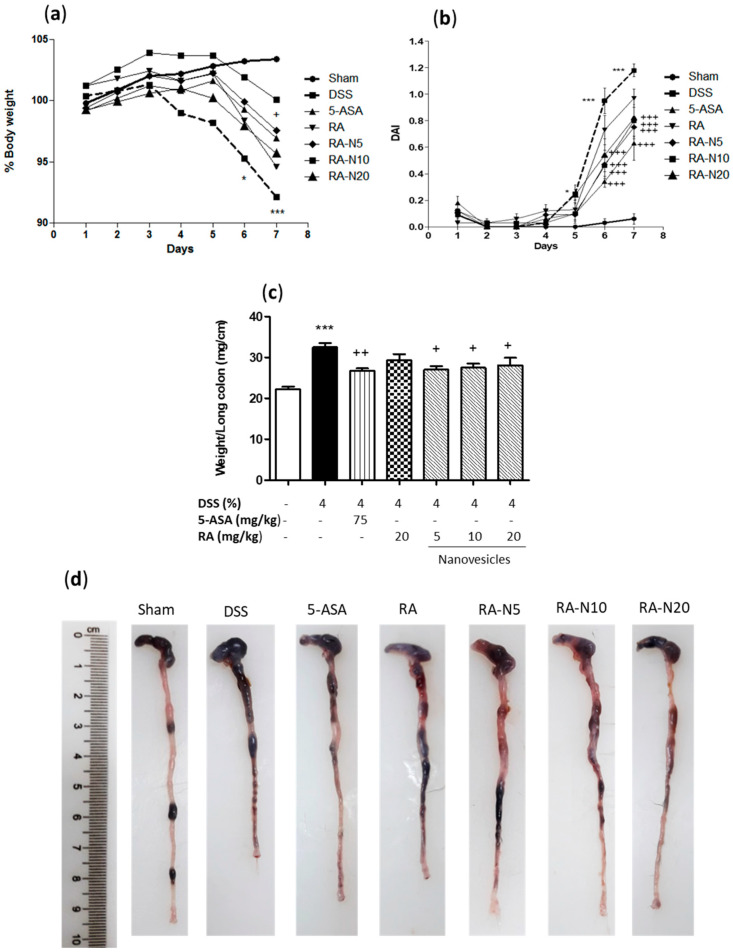
(**a**) Effects of rosmarinic acid (RA)-loaded nanovesicles (5, 10 and 20 mg/kg), RA (20 mg/kg) or aminosalicylic acid (5-ASA) (75 mg/kg) on body weight development in dextran sodium sulphate (DSS)-induced colitis in mice. Body weights were normalised against body weight on day –1. (**b**) Disease activity index (DAI) was determined as the average of the scores of bleeding, weight loss, and stool consistency. (**c**) Weight/length ratio of the colons. (**d**) Representative images of colons from all groups. Data are exhibited as the mean ± SEM. Mean value was significantly different compared with the sham group (* *p* < 0.05, *** *p* < 0.001; Student *t* test). Mean value was significantly different compared with DSS group (+ *p* < 0.05, ++ *p* < 0.01, +++ *p* < 0.01; one-way ANOVA followed by Bonferroni’s Multiple Comparison test).

It is known that DSS causes damage to the colonic epithelium, mimicking several aspects of UC [24]. As expected, the administration of drinking water ad libitum containing 4% (*w*/*v*) DSS, for seven days resulted in acute UC, characterised by a marked weight loss in relation to the sham group (Figure 2a). To evaluate the therapeutic effects of RA on acute colitis, RA-loaded nanovesicles were administered at the doses of 5, 10 and 20 mg/kg by oral route. Mice received pretreatment with nanovesicles from three days prior to the colitis induction and during days 1, 3, 5 and 7 of DSS administration. The positive control group was given 5-ASA at a dose of 75 mg/kg. Treatment with RA-loaded nanovesicles slightly increased body weight on day seven, being only significant with the dose of 10 mg/kg. Next, to assess the external signs of colitis, DAI score was evaluated (Figure 2b). This index showed no evidence of symptoms in sham animals. As expected, mice receiving DSS evidenced a significant increase in DAI score from the 5th day, reaching the peak on the 7th day. Free RA treatment failed to suppress the progression of colitis, resulting in a DAI index like that in the DSS group. However, administration of either 5-ASA or RA-loaded niosomes at all the doses assayed exhibited a marked decrease in DAI score from the 6th day, compared with DSS mice (*p* < 0.001). As shown in Figure 2c, a marked rise in colonic weight/length ratio, an indicator of colon inflammation, was found in the DSS group when compared with sham mice (*p* < 0.001). Free RA administration resulted in no significant changes in this parameter. Nevertheless, treatment with either 5-ASA or RA-loaded niosomes significantly attenuated colon inflammation (*p* < 0.01 and *p* < 0.05, respectively), as evidenced by the suppression of the weight/length ratio of the colon. According with these observations, macroscopic examination of the colons showed a colon shortening in DSS group, which was reversed following treatment with RA-loaded nanovesicles (Figure 2d). Altogether, these findings demonstrated that RA-loaded niosome administration substantially alleviated DSS‑induced colitis.

### 3.4. Rosmarinic Acid-Loaded Nanovesicles Administration Alleviated Microscopic Colon Damage and Increased Mucus Production

**Figure 3:**
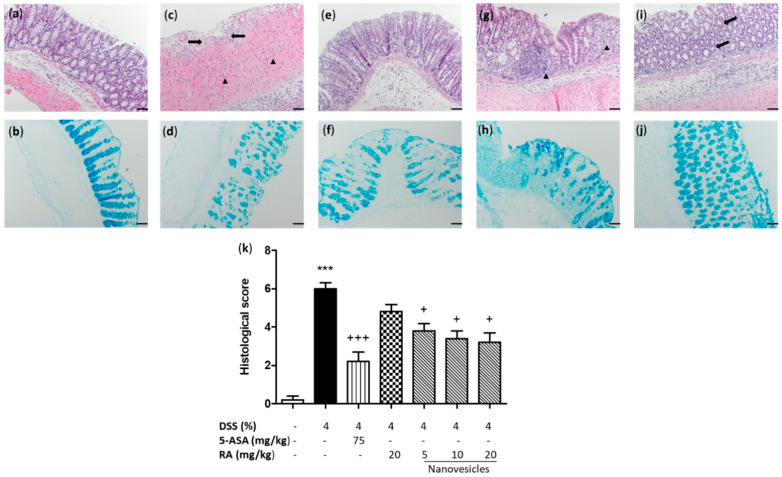
Rosmarinic acid (RA)-loaded nanovesicles administration attenuated DSS-induced microscopic colon damage and improved mucus accumulation inside the goblet cells. Histological images of colons following haematoxylin and eosin staining and Alcian blue staining: (**a**,**b**) sham group; (**c**,**d**) DSS group, ulceration (black arrows) and inflammatory cells (black triangle) in the lamina propria; (**e**,**f**) aminosalicylic acid (5-ASA) (75 mg/kg), (**g**,**h**) RA group (20 mg/kg), abundant inflammatory infiltrate (black triangle) and (**i**,**j**) RA-loaded nanovesicles (20 mg/kg, is shown as representative image of all the doses used), mucosal reparation and decrease of inflammatory infiltrate (black arrows). Original magnification 200 X. Scale bar: 50 µm. (**k**) Histopathological score of the colon was evaluated as indicated in the Methods section. Mean value was significantly different compared with the sham group (*** *p* < 0.001; Mann–Whitney U test). Mean value was significantly different compared with DSS group (+ *p* < 0.05, +++ *p* < 0.01; Kruskal–Wallis test).

The histopathological study of the colon of sham mice revealed a normal colonic appearance (Figure 3a). Consistent with macroscopic changes, the DSS group exhibited a higher inflammation score with mucosal damage, a massive inflammatory infiltrate (neutrophils, lymphocytes and histiocytes) mostly in the mucosa and submucosa and ulceration of the mucous epithelium (Figure 3c,k). Alcian blue staining, which reveals acid mucin positive goblet cells, displayed substantial mucin depletion near of the ulcerative areas of DSS-treated mice (Figure 3d) compared with sham group (Figure 3b). Free RA administration showed a slight improvement of microscopic signs of colitis and partial replacement of mucous secretion (Figure 3g,h). However, treatment with 5-ASA (Figure 3e) or RA-loaded nanovesicles revealed evident findings of mucosal reparation and a decrease of inflammatory infiltrate in the lamina propria with all the doses used in relation to DSS mice (Figure 3i, RA-loaded nanovesicles at 20 mg/kg) as well as a significant reduction of microscopic damage score (Figure 3k). Furthermore, Alcian blue-positive goblet cells were evident in the preserved areas of the mucosa after 5-ASA (Figure 3f) or RA-loaded nanovesicles treatment (Figure 3j).

### 3.5. Rosmarinic Acid-Loaded Nanovesicles Treatment Reduced Neutrophil Infiltration and Colonic TNF-α Production

**Figure 4:**
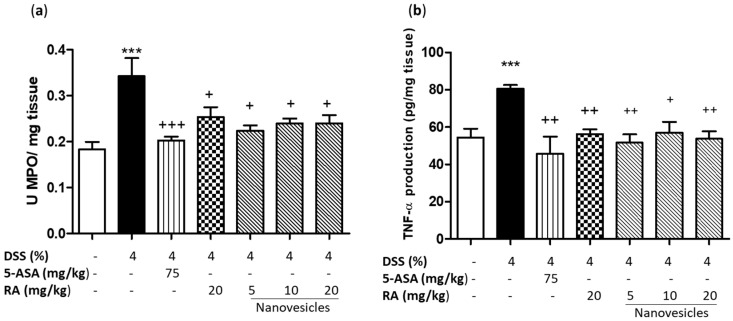
Effects of rosmarinic acid (RA)-loaded nanovesicles (5, 10 and 20 mg/kg), RA (20 mg/kg) or aminosalicylic acid (5-ASA) (75 mg/kg) on myeloperoxidase activity (**a**) and tumor necrosis factor alpha levels (**b**) in DSS-induced colitis in mice. Data are expressed as the mean ± SEM. Mean value was significantly different compared with the sham group (*** *p* < 0.001; Student *t* test). Mean value was significantly different compared with DSS group (+ *p* < 0.05, ++ *p* < 0.01, +++ *p* < 0.01; one-way ANOVA followed by Bonferroni’s Multiple Comparison test).

Neutrophil infiltration found in histological analysis of colons from DSS mice correlated with increased colonic MPO activity, a marker for inflammatory cell infiltration (*p* < 0.001 vs. sham group) (Figure 4a). As expected, 5-ASA treatment markedly reduced MPO activity in relation to the DSS group (*p* < 0.001). Similarly, this parameter was significantly decreased after treatment with free RA (20 mg/kg) or RA-loaded nanovesicles at all doses used (*p* < 0.05).

In addition, colonic damage by DSS administration was characterised by a marked increase in TNF-α levels in comparison with sham animals (*p* < 0.001). Administration of 5-ASA significantly reduced this cytokine production in relation to DSS mice (*p* < 0.01). Similar findings were detected following administration of free RA (20 mg/kg) (*p* < 0.01) or RA-loaded nanovesicles at 5, 10 and 20 mg/kg (*p* < 0.01, *p* < 0.05 and *p* < 0.01, respectively) (Figure 4b).

### 3.6. Rosmarinic Acid-Loaded Nanovesicles Administration Reduced Inflammasome Activation.

**Figure 5:**
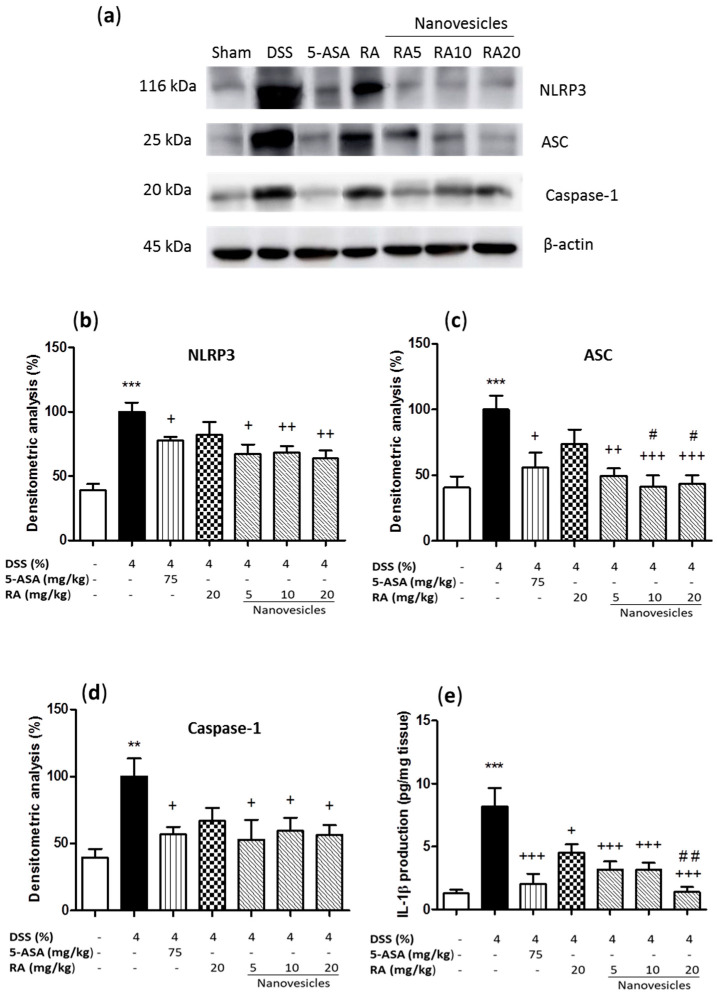
Effects of rosmarinic acid (RA)-loaded nanovesicles (5, 10 and 20 mg/kg), RA (20 mg/kg) or aminosalicylic acid (5-ASA) (75 mg/kg) on inflammasome components expression and IL-1β levels in DSS-induced colitis in mice. (**a**) Representative Western blot analysis of inflammasome proteins. Densitometry analysis of (**b**) nucleotide-binding domain, leucine-rich-repeat-containing family, pyrin domain- containing 3 (NLRP3), (**c**) inflammasome adaptor protein (ASC) and (**d**) caspase-1 were performed following normalization to the control (β-actin housekeeping gene). (**e**) IL-1 β production was evaluated by ELISA assay. Results are representative of five experiments performed on different simples. Data are expressed as the mean ± SEM. Mean value was significantly different compared with the sham group (** *p* < 0.01, *** *p* < 0.001; Mann–Whitney U test). Mean value was significantly different compared with DSS group (+ *p* < 0.05, ++ *p* < 0.01, +++ *p* < 0.001; Kruskal–Wallis test) or RA group (# *p* < 0.05, # # *p* < 0.01; Kruskal–Wallis test).

To support the beneficial effects of RA-loaded nanovesicles on acute colitis and investigate the potential action mechanisms, we studied the expression levels of different inflammasome-related proteins in colon samples. Our results evidenced that DSS administration led to upregulation of NLRP3, ASC and caspase-1 expression (Figure 5a–d) and, consequently, induced a significant increase in IL-1β levels in relation to sham animals (Figure 5e). Treatment with either 5-ASA or RA-loaded nanovesicles at all the doses used significantly downregulated the expression levels of inflammasome-related proteins. However, free RA administration did not induce significant changes in these proteins. Interestingly, significant differences in ASC protein expression were observed between RA-loaded nanovesicles at the doses of 10 and 20 mg/kg and free RA group (*p* < 0.05). As regards IL-1β production, administration of 5-ASA, free RA or RA-loaded nanovesicles resulted in a significant suppression of these cytokine levels as compared with DSS animals. Moreover, a marked difference was found in RA-loaded nanovesicles at 20 mg/kg in relation to free RA (*p* < 0.01).

### 3.7. Treatment with Rosmarinic Acid-Loaded Nanovesicles Increased Nrf-2 Antioxidant Signaling Pathway

**Figure 6:**
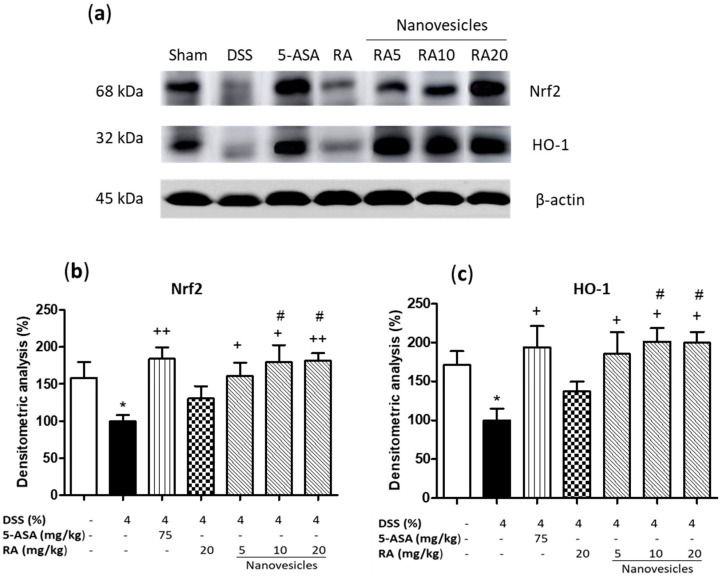
Effects of rosmarinic acid (RA)-loaded nanovesicles (5, 10 and 20 mg/kg), RA (20 mg/kg) or aminosalicylic acid (5-ASA) (75 mg/kg) on nuclear factor erythroid-related factor 2 (Nrf2) and heme oxygenase-1 (HO-1) expression in DSS-induced colitis in mice. (**a**) Representative Western blot analysis. Densitometry analysis of (**b**) Nrf2, and (**c**) HO-1 were performed following normalisation to the control (β-actin housekeeping gene). Results are representative of five experiments performed on different simples. Data are expressed as the mean ± SEM. Mean value was significantly different compared with the sham group (* *p* < 0.05; Mann–Whitney U test). Mean value was significantly different compared with DSS group (+ *p* < 0.05, ++ *p* < 0.01; Kruskal–Wallis test) or RA group (# *p* < 0.05; Kruskal–Wallis test).

To further explore the protective mechanism of RA-loaded nanovesicles, we investigated their ability to activate Nrf2 pathway, which stimulates the transcription of antioxidant genes and detoxification of enzymes such as HO-1 to protect against DSS-induced oxidative damage [5]. Our results reported that DSS prevented the increase of Nrf2 and HO-1 expression (Figure 6a–c). Administration of 5-ASA or RA-loaded nanovesicles at all the doses assayed significantly upregulated Nrf2 and HO-1 expression levels, reaching higher Nrf2 values that those in sham animals. Nevertheless, treatment with nonencapsulated RA did not induce significant changes in the expression of these antioxidant proteins. Remarkably, significant differences in Nrf2 and HO-1 levels were found between RA-loaded nanovesicles at 10 and 20 mg/kg and free RA (*p* < 0.05).

## 4. Discussion

IBD, including UC and CD, are chronic and recurrent disorders of the gastrointestinal tract [1]. Since current IBD treatments have limited results with many side effects, many researchers aimed to find new strategies for controlling symptoms and preventing relapses. The use of nutraceuticals with anti-inflammatory properties is gaining considerable attention for IBD treatment due to their safety. The anti-inflammatory effects of RA have been previously evidenced in different experimental models of inflammatory diseases, including arthritis, colitis and atopic dermatitis [15]. As regards colitis, the previous papers assayed higher doses of RA than those used in our study (25–200 mg/kg) and none of them evaluated the effects of RA on the modulation of the NLRP3 inflammasome or the antioxidant signaling pathway Nrf-2/HO-1 [25,32,33]. On the other hand, the poor water solubility and low bioavailability limited the clinical use of this polyphenol [23]. The application of nanosystems based on colon targeted drug delivery is receiving considerable attention for local treatment of UC since they can decrease drug loss in proximal intestine and thus increase drug concentration in the colon [34]. Conventional nanovesicles are scarcely used since they can suffer from gastric and enzymatic degradation, reducing the drug oral bioavailability. To overcome such limitation, chitosan is widely used for coating nanovesicles since this polymer resists gastric degradation, thus improving drug bioavailability. In this line, it has been previously reported that the recovery of phospholipid vesicles with chitosan and nutriose protected quercetin from upper gastrointestinal tract degradation, carrying it to the colon where the drug was released in inflamed tissue [20]. In the present study, we evaluated the effects of RA-loaded niosomes coated with the combination of chitosan and nutriose on DSS-induced colitis.

In terms of niosome characterisation, as expected our observations revealed that chitosan and nutriose coating increased the overall vesicle size. As regards the ZP study, a marked difference between uncoated and coated niosomes was observed. This parameter was negative for uncoated vesicles (approx. −17 mV) and an increase in the positive charges in the surface of chitosan-coated niosomes was observed (approx. +50 mV, data not shown), suggesting the adsorption of this positively-charged polysaccharide onto the niosomal surface. These positive charges were only partially neutralized by nutriose (approx. +38 mV), showing the formation of the polymeric complex by electrostatic interaction. In relation to in vitro drug release studies, uncoated and coated niosomes showed similar release profiles, which were significantly lower than that of the RA solution. Nevertheless, coated niosomes were selected for the present studies in order to protect nanovesicles from gastric degradation and increase colonic release [19].

Currently, DSS is widely used to induce experimental acute colitis since its mimics the clinical and histological characteristics of human UC [35]. In the present study, DSS induced colonic inflammation and mucosal damage, leading to body weight loss, colon shortening and increased DAI score. Treatment of mice with RA-loaded niosomes attenuated body weight loss and colon shortening, as well as relieved clinical symptoms of colitis. Furthermore, this formulation prevented histological damage by decreasing neutrophil infiltration and restoring epithelial cell injury. Intestinal mucus, synthesised by goblet cells, forms a gel-like layer that fills the crypts and serves as a barrier to protect the intestinal epithelium from the deleterious effects of luminal stimulants. Disruption of goblet cells can lead to intestinal inflammation [36]. It has been reported that IBD patients have reduced numbers of goblet cells and mucus layer thickness [37]. Our study exhibited mucin-depleted crypts in colitic mice, as evidenced by the loss of Alcian blue-stained goblet cells. Remarkably, RA-loaded nanovesicles administered orally enhanced mucus accumulation inside the goblet cells, suggesting a protective role of this formulation on colonic epithelial damage.

It has been reported that inflammatory cell infiltration into colon tissue has a pathogenic role in IBD. Therefore, its control is very important for the attenuation of this disease. MPO activity serves as a marker for measuring neutrophil inflammatory response after colitis induction. Once released, this enzyme catalyses the production of ROS, which are involved in IBD development. In addition, immune cells release pro-inflammatory cytokines such as TNF-α, which is involved in the initiation and maintenance of mucosal inflammation in IBD [27]. Our data evidenced that free RA or RA-loaded nanovesicles effectively inhibited polymorphonuclear infiltration into the colon, as shown by reduced colonic MPO activity and, consequently, decreased colonic TNF-α levels. In according with this findings, the anti-inflammatory effects of RA, mediated by reduction of TNF-α release, have been previously reported in experimental colitis [25,32]. Moreover, a recent paper showed that PEGylated RA-derived nanoparticles (10, 20 and 30 mg/kg) inhibited TNF-α production dose-dependently in DSS-induced colitis [23].

The NLRP3 inflammasome is the most extensively investigated inflammasome complex and is composed of NLRP3 protein, ASC adaptor and caspase-1. This last protein can eventually produce the maturation and release of IL-1β [3]. In previous studies, RA was shown to inhibit inflammasome activation in experimental models of inflammation in epidermal keratinocytes [38], neuroinflammatory injury [39], atherosclerosis [40] and premature ovarian failure [41]. Despite these findings, there is no previous evidence that RA may exert its anti-inflammatory effects through suppression of inflammasome activation in UC. In the present study, we reported for the first time that orally-administered RA-loaded niosomes markedly reduced inflammasome-related proteins such as NLRP3, ASC and caspase-1 in a DSS-induced colitis experimental model in mice. However, this effect was not detected with the parent compound. In terms of IL-1β production, the colonic levels of this cytokine were decreased by both free RA and RA-loaded niosomes. Since nonencapsulated RA did not significantly modify inflammasome-associated proteins expression, the reduced levels of IL-1β after RA treatment could be alternatively explained by the inhibitory effect of RA on NF-kB activation, as previously reported [25,42].

Although the mechanism underlying UC is not clear, oxidative stress has been reported to play an important role in colitis pathogenesis. Inflammatory cells produce high ROS levels, which lead to oxidative damage in the colon. Nrf2 is a transcription factor that modulates cellular antioxidant response. Under oxidative conditions, this cytoplasmic factor is translocated into the nucleus where it reacts with the antioxidant response element (ARE) and induces transcription of antioxidant genes such as HO-1 [5]. RA has been previously reported to activate Nrf2/HO-1 signaling pathway in different experimental models including spinal cord injury [43], high-fat diet-induced intestinal damage [44], acute liver injury [45] and streptozotocin-induced diabetes [46]. In the present study, our data evidenced that DSS decreased Nrf2 protein expression and its targeted gene HO-1, in comparison to the sham group. The levels of both proteins were reverted by treatment with the positive control 5-ASA and RA-loaded niosomes. However, oral administration of nonencapsulated RA did not induce significant changes in the expression levels of these antioxidant proteins. In agreement with these observations, a recent in vitro study by our group reported that the treatment of UVB-exposed HaCaT cells with RA alone had no significant effects on Nrf2/HO-1 pathway regulation. However, the combination of the carotenoid fucoxanthin and RA protected against UVB-induced oxidative stress through upregulation of Nrf2 transcriptional factor and its main target gene HO-1 [47].

In the present study, it is noteworthy that the nonencapsulated RA treatment was only able to significantly reduce MPO activity and TNF-α levels, early markers for acute inflammation. However, the RA-free treatment did not induce significant changes in regulation of NLRP3 inflammasome or the Nrf2/HO-1 signaling pathway in relation to niosomes treatment. The loss of biological activity of free RA could be explained by its gastric degradation and metabolization in the upper gastrointestinal tract. In this regard, the use of chitosan and nutriose-coated niosomes loaded with RA may be a useful alternative to nonencapsulated RA because these formulations could increase RA local bioavailability and allow a controlled release in the inflamed colon. As a consequence, lower doses of RA could be used, which would reduce production cost for a possible future application in the treatment of patients with colitis.

## 5. Conclusions

Our findings indicate that chitosan and nutriose-coated niosomes loaded with RA showed a beneficial effect in acute experimental colitis, with all the doses being effective, although a dose-response relation was not detected. Our study is the first to show that these RA nanovesicles could protect the colonic mucosa against DSS-induced damage by attenuating inflammation and oxidative stress through modulation of NLRP3 inflammasome and reestablishment of the Nrf2/HO-1 signaling pathway. Therefore, this formulation could be a novel nutraceutical approach to oral colon-targeted UC therapy. However, further studies in a chronic model of colitis are needed in order to deepen the dose-response effect and the pharmacokinetic profile of these RA-loaded niosomes.

## Figures and Tables

**Table 1:** Average size, zeta potential and encapsulation efficiency of uncoated niosomes loaded with rosmarinic acid (Nio-RA), empty chitosan and nutriose-coated niosomes (CN-Nio) and chitosan and nutriose-coated niosomes loaded with RA (CN-Nio-RA).

Sample	Size (nm)	Zeta Potential (mv)	Encapsulation Efficiency (%)
Nio-RA	260.7 ± 6.1	−17.2 ± 0.5	73.7 ± 0.7
CN-Nio	429.7 ± 11.2	+24.3 ± 1.0	-
CN-Nio-RA	480.5 ± 15.8	+38.8 ± 1.2	73.7 ± 0.7

## Data Availability

The data presented in this study are available from the corresponding author (E.T.) upon reasonable request.
